# Epidemiological characteristics of hospitalized pediatric patients with acute respiratory tract infection during and after the COVID-19 period in Ningbo, China: a retrospective single-center study

**DOI:** 10.1186/s12879-026-13316-4

**Published:** 2026-05-23

**Authors:** Bibo Mao, Daina Chen, Zhuoling Li, Wenbo Lu, Wenyuan Liu

**Affiliations:** 1 Laboratory Department, The Affiliated Women and Children’s Hospital of Ningbo University, Ningbo, China; 2 Pediatrics Department, The Affiliated Women and Children’s Hospital of Ningbo University, Ningbo, China; 3 Ningbo Key Laboratory for the Prevention and Treatment of Embryo Original Diseases, The Affiliated Women and Children’s Hospital of Ningbo University, Ningbo, China; 4 Ningbo Key Laboratory of Genomic Medicine and Birth Defects Prevention, The Affiliated Women and Children’s Hospital of Ningbo University, Ningbo, China

**Keywords:** COVID-19, Children, Epidemiology, Acute respiratory infection

## Abstract

**Background:**

This study aimed to investigate the changes in the epidemiological characteristics of respiratory pathogens among hospitalized children in Ningbo between the COVID-19 pandemic period (2020–2022) and the post-pandemic period (2023–2024), to provide scientific data for formulating effective prevention and control strategies for respiratory infections.

**Methods:**

A total of 59,526 pediatric patients hospitalized for acute respiratory infections at the Ningbo University Affiliated Women and Children’s Hospital from January 2020 to December 2024 were enrolled. Throat swab samples were collected, and 11 respiratory pathogens were detected using multiplex reverse transcription-polymerase chain reaction (RT-PCR) combined with capillary electrophoresis. The distribution of respiratory pathogens was analyzed across different genders, age groups, and seasons.

**Results:**

Among the enrolled children, human rhinovirus (HRV) was the most frequently detected pathogen (17.69%), followed by *Mycoplasma pneumoniae* (Mp, 17.45%) and human respiratory syncytial virus (HRSV, 8.46%). Joinpoint analysis revealed four significant turning points in monthly pathogen positivity rates in April 2020, September 2020, January 2023, and October 2023. The average pathogen positivity rate increased from 49.06% during the pandemic to 69.13% in the post-pandemic period. Specifically, the positivity rates of influenza A (FluA), influenza B (FluB), human adenovirus (HAdV), and Mp increased, while those of human bocavirus (HBoV), HRV, and HRSV decreased. No significant changes were observed for human parainfluenza virus (HPIV), *Chlamydia* (Ch), human metapneumovirus (HMPV), and human coronavirus (HCoV). The positivity rate was consistently higher in males than in females throughout the study period (*P <* 0.05). The Mp positivity rate increased with age (χ²=6291.100, *P <* 0.001), whereas the HRSV positivity rate decreased with age (χ²=2377.650, *P <* 0.001). Seasonal increases in detection were observed: Mp positivity rates increased markedly from April 2023, peaking at 50%; FluA detection increased from March to May 2023 and October 2023 to May 2024; FluB circulated from November 2023 to April 2024; and a HAdV surge began in May 2024. The rate of mixed infections increased from 5.76% to 13.69%.

**Conclusion:**

Significant shifts in the detection patterns of respiratory pathogens occurred in the post-pandemic period compared to the pandemic period, marked by increased overall pathogen positivity rates among hospitalized children and mixed infection rates. Successive increases in the positivity rates of FluA, FluB, Mp, and HAdV were identified after the pandemic.

**Clinical trial number:**

Not applicable.

## Background

Acute respiratory infections (ARIs) are a leading cause of pediatric hospitalization and a major threat to children’s health globally. Annually, children may experience 3–8 episodes of ARI, imposing a substantial burden on healthcare systems [1, 2]. Symptoms range from cough, fever, and runny nose to general malaise. ARIs can manifest as mild, self-limiting upper respiratory tract infections or progress to severe lower respiratory tract infections such as pneumonia and bronchiolitis, potentially leading to death [3]. The causative agents include bacteria, viruses, and mycoplasma, which can cause single or mixed infections. Due to their relatively immature immunity, children are particularly susceptible to mixed infections [4, 5]. Common pathogens in recent years include influenza virus, *Mycoplasma pneumoniae* (Mp), human respiratory syncytial virus (HRSV), and human adenovirus (HAdV) [6, 7].

The COVID-19 pandemic presented unprecedented challenges to global public health and heightened awareness of respiratory disease threats [8]. In response, China and other countries implemented various non-pharmaceutical interventions (NPIs) such as mask-wearing, hand hygiene, social distancing, and lockdowns to control SARS-CoV-2 transmission [9, 10]. While these measures curbed the spread of SARS-CoV-2, they also altered the circulation of other respiratory pathogens. Reduced exposure may have led to a decline in population immunity, potentially increasing susceptibility to these pathogens upon resurgence [11, 12]. Following the announcement of the “Ten Measures” policy in early December 2022, China entered a post-pandemic phase characterized by heightened activity of multiple respiratory pathogens. In 2023, northern and southern China experienced rapid surges in infections caused by pathogens like Mp and HRSV [13–15]. In 2024, the positivity rate of HAdV has risen rapidly [16]., indicating a shift in epidemic patterns. Consequently, continuous monitoring of the epidemiological characteristics of these pathogens has become crucial.

China’s vast territory encompasses diverse climates, and the prevalence of respiratory pathogens is influenced by climate, seasonality, and population density [17, 18]. Ningbo, located on the southeastern coast with a subtropical monsoon climate and a population exceeding nine million, represents a significant urban center. To date, no analysis has been conducted on the epidemiological characteristics of respiratory pathogens among hospitalized children in this region following the end of the dynamic zero-COVID policy. This study retrospectively analyzed laboratory data from 59,526 hospitalized children at the Ningbo University Affiliated Women and Children’s Hospital from January 2020 to December 2024. It aims to examine changes in respiratory pathogen prevalence between the pandemic (2020–2022) and post-pandemic (2023–2024) periods, providing evidence to inform future prevention and control strategies.

## Methods

### Study subjects and data sources

This retrospective study enrolled patients diagnosed with acute respiratory infections (ARI) who were admitted to the pediatric wards of Ningbo University Affiliated Women and Children’s Hospital from January 1, 2020, to December 31, 2024. Inclusion criteria were: (1) children meeting the diagnostic criteria for respiratory tract infections as outlined in “Zhu Futang’s Practical Pediatrics“ [19], which includes: (a) acute upper respiratory tract infection: presence of nasal congestion, rhinorrhea, sore throat, and/or cough with or without fever, with symptom duration of less than 10 days; (b) acute lower respiratory tract infection (bronchitis): cough as the predominant symptom, with or without sputum production, accompanied by auscultatory findings of wheezing or coarse breath sounds; (c) pneumonia: presence of fever, cough, tachypnea, and abnormal auscultatory findings (rales or decreased breath sounds), with radiological confirmation of pulmonary infiltrates; (2) aged 0–17 years; (3) having undergone testing for 11 respiratory pathogens. Exclusion criteria were: (1) Incomplete or missing data: missing age, sex, admission date, or pathogen test results; (2) Primary immunodeficiency disorders: including but not limited to severe combined immunodeficiency (SCID), common variable immunodeficiency (CVID), X-linked agammaglobulinemia, Wiskott-Aldrich syndrome, DiGeorge syndrome, or other genetically confirmed primary immunodeficiencies; (3) Secondary immunodeficiency: HIV infection (confirmed by serology or PCR), immunosuppressive therapy within the past 3 months (systemic corticosteroids ≥ 0.5 mg/kg/day prednisone equivalent for ≥ 14 days, chemotherapy, radiation therapy, biologic agents, or other immunosuppressive medications), post-organ transplantation status, or splenectomy; (4) Hematologic malignancies: leukemia, lymphoma, or other hematologic malignancies at any stage, whether active or in remission; (5) Solid malignancies: active solid tumors requiring chemotherapy, radiation therapy, or surgical intervention within the past 6 months; Relevant data were extracted from the hospital’s electronic medical records, including demographic information (age, sex), admission dates, and pathogen test results. Detailed clinical data such as disease severity scores, intensive care unit admission, length of hospital stay, and mortality were not systematically collected. This study was conducted in accordance with the Declaration of Helsinki and was approved by the Ethics Committee of The Affiliated Women and Children’s Hospital of Ningbo University (Approval No. NBFE-2025-KY-207). As a retrospective analysis of anonymized data, the requirement for informed consent was waived by the Ethics Committee.

### Laboratory testing

Throat swab samples were collected by depressing the child’s tongue with a tongue depressor and rubbing a flocked swab against the posterior pharyngeal wall and both tonsils. The swab was then placed into 3 mL of cell preservation solution.

Multiplex RT-PCR combined with capillary electrophoresis was used to detect 11 respiratory pathogens: influenza A virus (FluA), influenza B virus (FluB), HAdV, human parainfluenza virus (HPIV), human rhinovirus (HRV), human metapneumovirus (HMPV), human coronavirus (HCoV), human bocavirus (HBoV), HRSV, Mp and *Chlamydia* (Ch)(including *Chlamydia trachomatis* and *Chlamydia pneumoniae*), Nucleic acid extraction and multiplex PCR kits (National Medical Device Approval No. 20183400518) were purchased from Ningbo Health Gene Technologies Company Limited. Procedures were performed strictly according to the manufacturer’s instructions. Each run included positive and negative controls. Specimen quality was monitored using human RNA and DNA internal references. A result was considered positive if characteristic pathogen peaks were detected alongside internal reference peaks [20, 21].

### Statistical analysis

Statistical analyses were performed using SPSS (version 26.0). Normally distributed measurement data are presented as mean ± standard deviation, while categorical data are expressed as counts or percentages. Intergroup comparisons were made using the χ2 test or Fisher’s exact test, as appropriate. Trends in positivity rates were analyzed using the χ2 trend test. Temporal trends in overall monthly pathogen activity were analyzed using Joinpoint regression analysis (Joinpoint software, version 5.4.0; National Cancer Institute, Bethesda, MD, USA) [22]. This method identifies statistically significant inflection points (joinpoints) where the slope of the trend changes, allowing for the characterization of temporal patterns through piecewise linear regression. Model selection was based on the Bayesian Information Criterion (BIC) and permutation tests, with the final model representing the best fit with the smallest number of joinpoints that significantly improved model fit. The monthly percentage change (MPC) was calculated with 95% confidence intervals to quantify trend magnitudes. A two-tailed P value of less than 0.05 was considered statistically significant.

## Results

### General characteristics and detection rates of the study population

A total of 59,526 hospitalized children with ARI were included: 26,476 from the pandemic period (2020–2022) and 33,050 from the post-pandemic period (2023–2024). Participants were categorized into four age groups: infants (0–<1 year, *n* = 13,460), toddlers (1–<2 years, *n* = 7,522), preschool children (2–<6 years, *n* = 21,829), and adolescents (6–17 years, *n* = 16,715). The number of cases increased in both genders during the post-pandemic period. The positivity rates of FluA, FluB, HAdV, and Mp increased significantly (*P* < 0.05), while those of HBoV, HRV, and HRSV decreased (*P* < 0.05). No significant differences were observed for HPIV, Ch, HMPV, and HCoV (*P* > 0.05) (Table 1).

**Table 1:** The pathogen detection rates during and post the pandemic periods [cases (%)]

Variable	During COVID-19			Post COVID-19				Overall *n* = 59,526	χ²	*P*
	2020 *n* = 7995 *n* = 7995	2021 *n* = 9027	2022 *n* = 9454	Overall *n* = 26,476	2023 *n* = 17,512	2024 *n* = 15,538	Overall *n* = 33,050			
Gender										
Male	2396(52.67)	2857(55.25)	2364(44.54)	7617(50.69)	6362(67.06)	6072(72.05)	12,434(69.41)	20,051(60.87)	1201.927	< 0.001
Female	1710(49.62)	1966(50.99)	1695(40.87)	5371(46.91)	5338(66.52)	5075(71.38)	10,413(68.80)	15,784(59.37)	1294.665	< 0.001
Age groups										
0 ~ < 1year	1157(48.90)	1412(49.70)	862(34.19)	3431(44.40)	1518(51.65)	1707(61.12)	3225(56.26)	6656(49.45)	185.377	< 0.001
1 ~ < 2years	777(55.78)	957(61.82)	446(42.12)	2180(54.50)	1179(65.57)	1215(70.48)	2394(67.97)	4574(60.81)	142.650	< 0.001
2 ~ < 6years	1720(58.88)	2055(57.72)	1896(47.77)	5671(54.27)	4409(71.00)	3998(77.35)	8407(73.88)	14,078(64.49)	915.119	< 0.001
6 ~ 17years	452(34.37)	399(37.01)	855(44.88)	1706(39.69)	4594(69.98)	4227(72.23)	8821(71.04)	10,527(62.98)	1345.617	< 0.001
Pathogen										
FluA	157(1.96)	4(0.04)	236(2.50)	397(1.50)	1676(9.57)	608(3.91)	2284(6.91)	2681(4.50)	1000.783	< 0.001
HAdV	313(3.91)	365(4.04)	491(5.19)	1169(4.42)	564(3.22)	2170(13.97)	2734(8.27)	3903(6.56)	356.924	< 0.001
HBoV	143(1.79)	234(2.59)	201(2.13)	578(2.18)	332(1.90)	147(0.95)	479(1.45)	1057(1.78)	45.381	< 0.001
HRV	1868(23.36)	2260(25.04)	1038(10.98)	5166(19.51)	2593(14.81)	2772(17.84)	5365(16.23)	10,531(17.69)	108.543	< 0.001
HPIV	533(6.67)	491(5.44)	349(3.69)	1373(5.19)	870(4.97)	872(5.61)	1742(5.27)	3115(5.23)	0.214	0.644
Ch	82(1.03)	59(0.65)	60(0.63)	201(0.76)	85(0.49)	180(1.16)	265(0.80)	466(0.78)	0.344	0.557
HMPV	286(3.58)	408(4.52)	532(5.63)	1226(4.63)	697(3.98)	882(5.68)	1579(4.78)	2805(4.71)	0.707	0.4
FluB	219(2.74)	217(2.40)	133(1.41)	569(2.15)	163(0.93)	1022(6.58)	1185(3.59)	1754(2.95)	106.050	< 0.001
Mp	255(3.19)	165(1.83)	765(8.09)	1185(4.48)	5416(30.93)	3786(24.37)	9202(27.84)	10,387(17.45)	5572.072	< 0.001
HCoV	200(2.50)	90(1.00)	74(0.78)	364(1.37)	210(1.20)	225(1.45)	435(1.32)	799(1.34)	0.382	0.537
HRSV	681(8.52)	1251(13.86)	489(5.17)	2421(9.14)	1457(8.32)	1158(7.45)	2615(7.91)	5036(8.46)	28.805	< 0.001

### Monthly distribution characteristics of respiratory pathogens

**Fig. 1:**
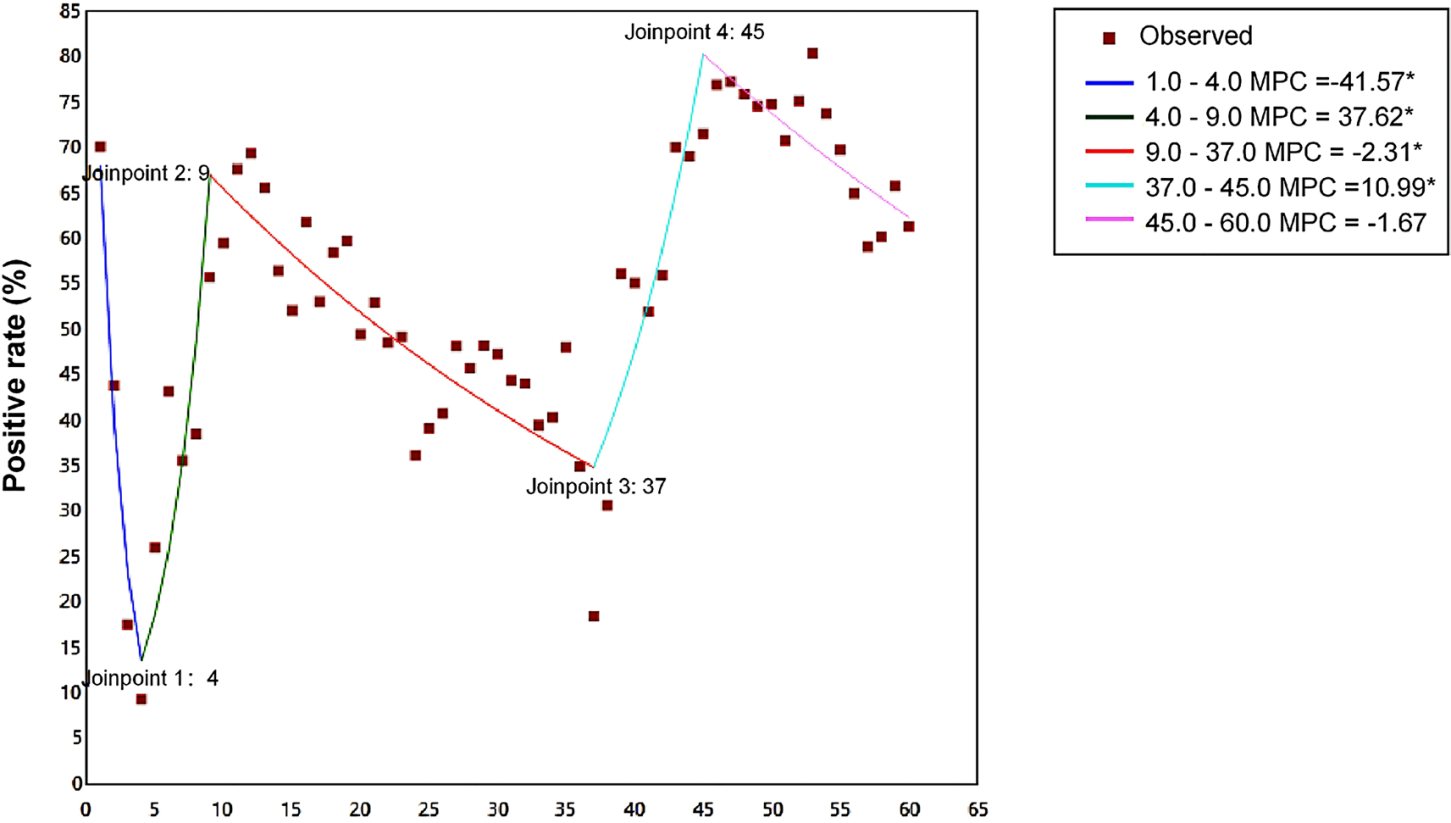
Joinpoint analysis of the monthly pathogen positivity rate from January 2020 to December 2024. The fitted model is shown by the colored line. The legend provides the Monthly Percentage Change (MPC) values. * indicates a significant difference from zero at the alpha = 0.05 level

The monthly positivity rate for any respiratory pathogen was analyzed using Joinpoint regression. Four significant turning points were identified in April 2020, September 2020, January 2023, and October 2023 (Fig. 1). The positivity rate sharply declined from January to April 2020 (MPC = -41.57%), rose rapidly until September 2020 (MPC = 37.62%), gradually declined until January 2023 (MPC = -2.31%), increased again until September 2023 (MPC = 10.99%), and then slowly declined until December 2024 (MPC = 1.67%). The average positivity rate was 49.06% during the pandemic and increased to 69.13% in the post-pandemic period.

### Gender-related characteristics of respiratory pathogens

**Fig. 2:**
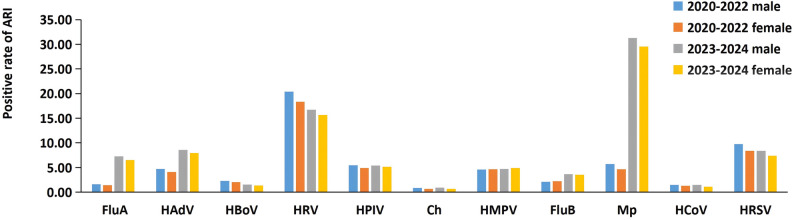
Positivity rates of ARI by gender, comparing the pandemic (2020–2022) and post-pandemic (2023–2024) periods

Positivity rates and case numbers increased for both genders in the post-pandemic period. However, males consistently had a significantly higher overall positivity rate than females in both periods (*P* < 0.05). During the pandemic period, the analysis of positive rates across different genders revealed that HRV had the highest positive rates in both males(20.41%) and females (18.33%).Notably, there was no statistically significant difference in the positive rates of FluA, HBoV, Ch, HMPV, FluB, Mp, and HCoV between males and females (*P* > 0.05), while the positive rate of HAdV and HPIV in males was slightly higher than that in females(*P* < 0.05), and the positive rates of HRV and HRSV were significantly higher in males compared to females(*P* < 0.05).In the post-pandemic period, we observed that the positive rates of FluA, HAdV, HRV, Ch, Mp, HCoV, and HRSV in males were higher than those in females, while there were no statistically significant differences in the positive rates of HBoV, HPIV, HMPV, and FluB between males and females (Fig. 2).

### Age-related characteristics of respiratory pathogens

**Fig. 3:**
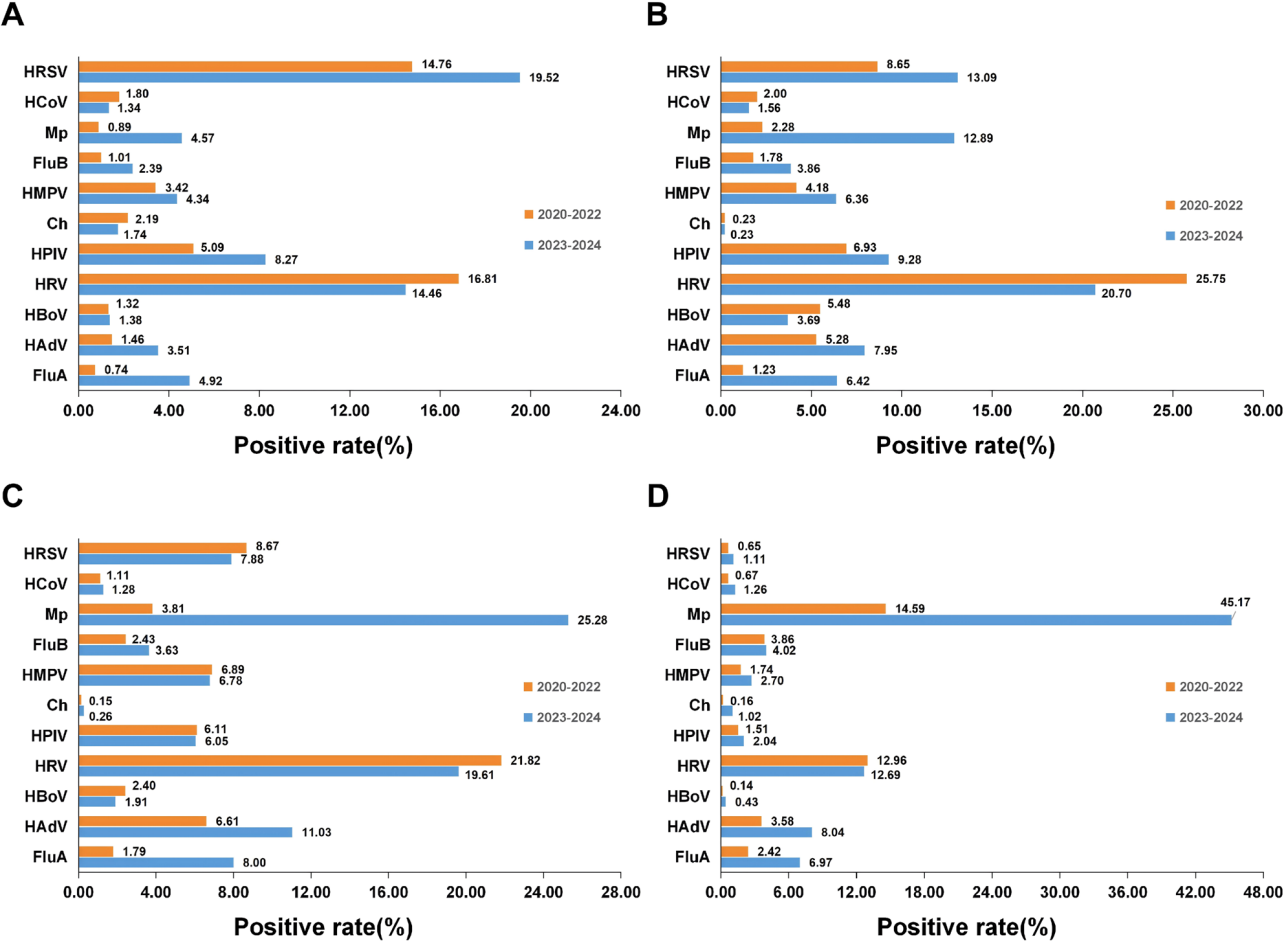
Positivity rates of ARI by age group, comparing the pandemic (2020–2022) and post-pandemic (2023–2024) periods. (**A**) Infants (0 ~ < 1 year), (**B**) Toddlers (1 ~ < 2 years), (**C**) Children (2 ~ < 6 years), (**D**) Adolescents (6 ~ 17 years)

During the pandemic, HRV had the highest positivity rate across all age groups except adolescents (6–17 years). In the post-pandemic period, both sample sizes and detection rates increased across all age groups (Table 1; Fig. 3). Pathogen distribution patterns shifted significantly with age.

In infants (0 ~ < 1 year), positivity rates for HRSV, Mp, FluB, HMPV, HPIV, HAdV, and FluA increased significantly (*P* < 0.05), with HRSV, HRV, and HPIV being the most common (Fig. 3A). In toddlers (1 ~ < 2 years), the positive rates of HRSV, Mp, FluB, HMPV, HPIV, HAdV, and FluA also exhibited a significant rise (*P* < 0.05), with HRV, HRSV, and Mp being most frequent (Fig. 3B).

Among preschool children (2 ~ < 6 years), positivity rates for Mp, FluB, HAdV, and FluA increased, while HRSV and HRV rates decreased. The top pathogens were Mp, HRV, and HAdV (Fig. 3C). In adolescents (6–17 years), all pathogen positivity rates except HRV increased significantly, with Mp, HRV, and HAdV being most common (Fig. 3D).

Chi-square trend analysis confirmed that the Mp positivity rate increased with age (χ²=6 291.100, *P* < 0.001), while the HRSV positivity rate decreased with age (χ²=2 377.650, *P* < 0.001).

### Season-related characteristics of respiratory pathogens

**Fig. 4:**
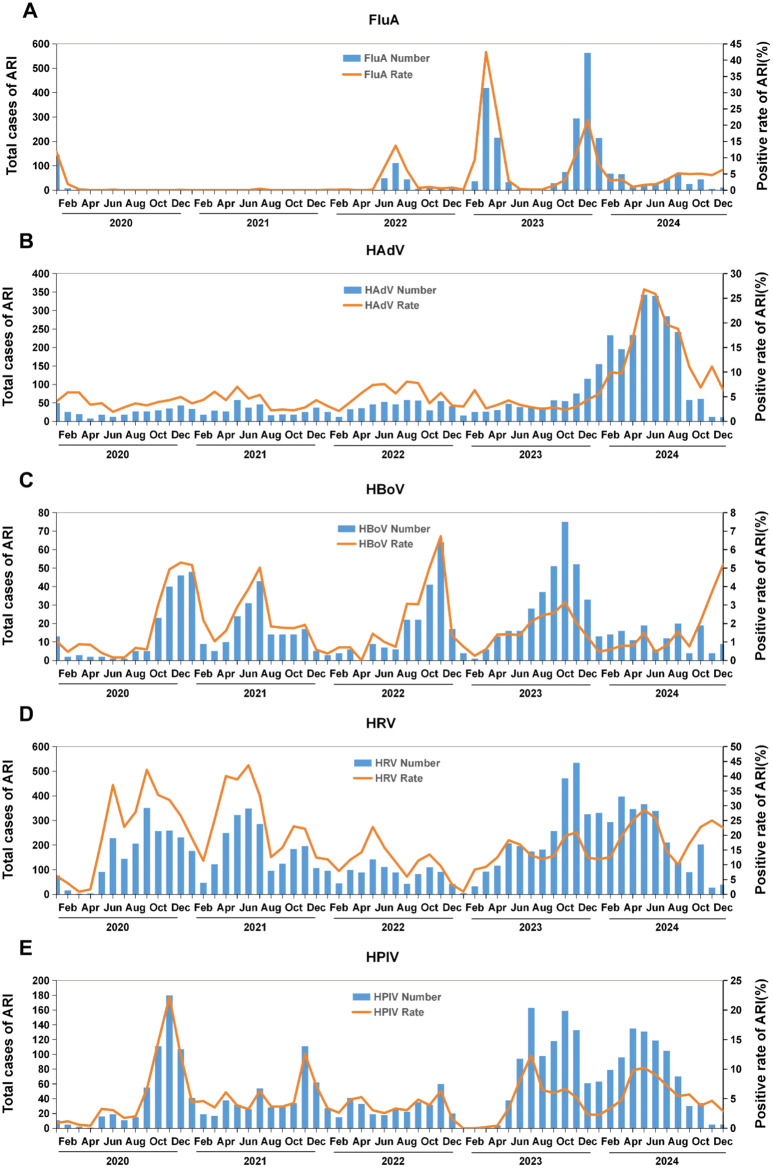

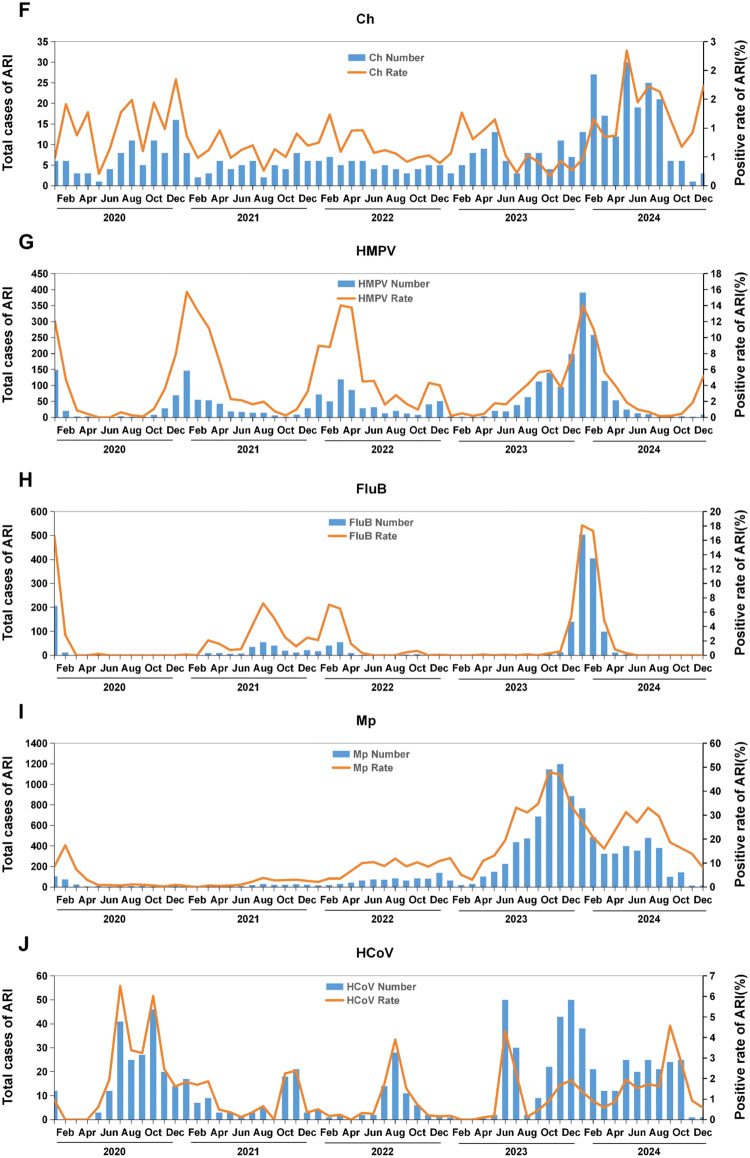

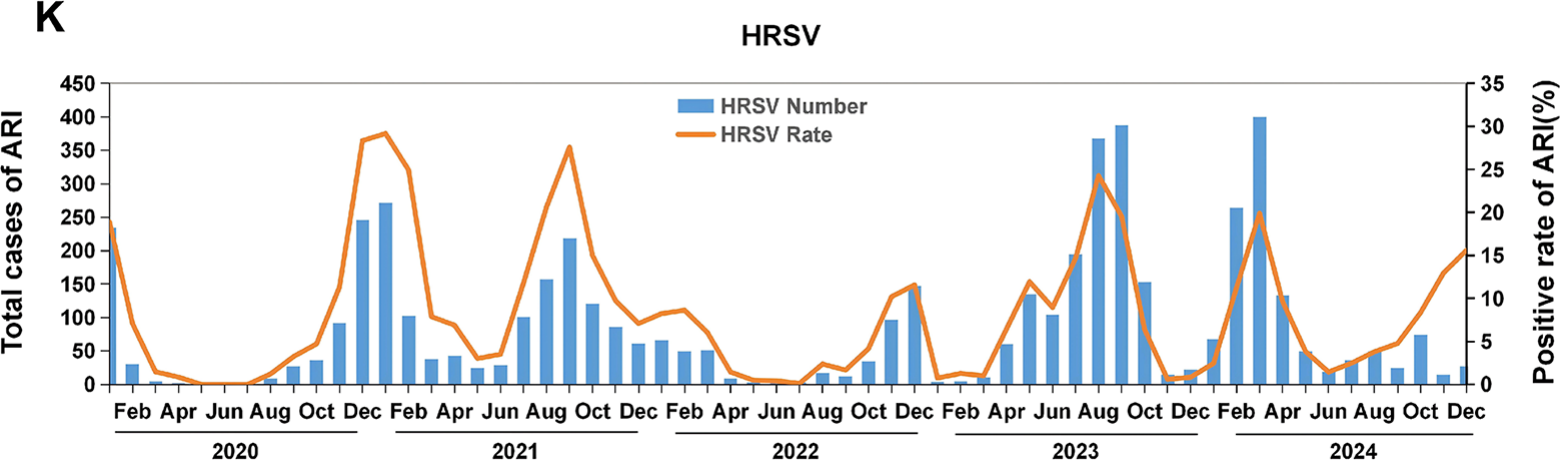
Seasonal trends of ARI positivity rates from January 2020 to December 2024. (**A**) Influenza A virus; (**B**) Human Adenovirus; (**C**) Human Bocavirus; (**D**) Human Rhinovirus; (**E**) Human Parainfluenza virus; (**F**) *Chlamydia*; (**G**) Human Metapneumovirus; (**H**) Influenza B virus; (**I**) *Mycoplasma pneumoniae*; (**J**) Human Coronavirus; (**K**) Respiratory syncytial virus

During the post-pandemic period, positivity rates for most pathogens increased, except for Ch, which remained consistently low (Fig. 4F).

During the pandemic period, FluA was nearly undetectable, with only a minor peak observed from May to September 2022, while outbreaks occurred from March to May 2023 and October 2023 to May 2024, reaching a positive rate as high as 43%(Fig. 4A). FluB was almost undetectable in 2020 but re-emerged after May 2021, with an outbreak occurring from November 2023 to April 2024 that reached an 18% positive rate(Fig. 4H).

HAdV activity was low during the pandemic but increased significantly from May 2024 (Fig. 4B). HBoV showed annual peaks around November, with an off-season peak in July 2021 (Fig. 4C). HRV rebounded first in June 2020, maintaining a high detection rate until November when it declined, subsequently showing two annual peaks from April to June and October to December(Fig. 4D).

HPIV showed annual winter peaks during the pandemic, with off-season peaks in May and July 2023 post-pandemic (Fig. 4E). HMPV typically peaked in winter but was delayed to March 2022 and September 2023 (Fig. 4G).

Mp saw its positive rate drop to nearly zero during the pandemic, maintaining low detection rates from April 2022 onward. In the post COVID-19 period, MP positive rates surged significantly starting April 2023, reaching up to 50% (Fig. 4I). HCoV exhibited low positive rates in both phases but showed minor peaks in July or September(Fig. 4J). HRSV primarily peaked during autumn and winter seasons, while off-season peaks occurred from May to September 2023 and February to April 2024 in the post-pandemic period(Fig. 4K).

### Single infection and co-infections characteristics of respiratory pathogens

During the pandemic period, 11,463 out of 26,476 cases (43.30%) were infected with a single pathogen, while 1,525 cases (5.76%) were infected with two or more pathogens. These infection rates were significantly lower than those in the post-pandemic period, where the single infection rate was 55.44% and the mixed infection rate was 13.69% (*P* < 0.05). The infection rates of dual, triple, and quadruple infections were also significantly lower than those in the post-pandemic period (*P* < 0.05) (Table 2).

During the pandemic period, 1,397 cases (5.28%) of mixed infections were dual infections, with the most common being HRV+HRSV at 219 cases (15.68%), followed by HRV+HPIV at 209 cases (14.96%) and HRV+HADV at 155 cases (11.10%). There were 120 cases of triple infections and 8 cases of quadruple infections. In the post-pandemic period, 4,054 cases (12.27%) of mixed infections were dual infections, with the most common being HRV + Mp at 1,045 cases (25.78%), followed by HRV+HADV at 369 cases (9.10%) and HADV + Mp at 261 cases (6.44%). There were 431 cases of triple infections, 38 cases of quadruple infections, and 2 cases of quintuple infections, specifically HRV+HPIV + Mp+HRSV+HBoV and HADV+HBoV+HPIV + Mp+HRSV.

**Table 2:** The single infection and co-infections of respiratory pathogens from 2020 to 2024 [cases (%)]

Variable	During COVID-19				Post COVID-19			χ2	*P*
	2020 *n* = 7995	2021 *n* = 9027	2022 *n* = 9454	Overall *n* = 26,476	2023 *n* = 17,512	2024 *n* = 15,538	Overall *n* = 33,050		
Single infection	3539(44.27)	4159(46.07)	3765(39.82)	11,463(43.30)	9566(54.63)	8756(56.35)	18,322(55.44)	866.692	< 0.001
Dual infection	508(6.35)	610(6.76)	279(2.95)	1397(5.28)	1928(11.01)	2126(13.68)	4054(12.27)	863.348	< 0.001
Triple infection	54(0.68)	51(0.56)	15(0.16)	120(0.45)	185(1.06)	246(1.58)	431(1.30)	116.041	< 0.001
Quadruple infection	5(0.06)	3(0.03)	0(0.00)	8(0.03)	19(0.11)	19(0.12)	38(0.11)	12.677	< 0.001
Five-fold infection	0(0.00)	0(0.00)	0(0.00)	0(0.00)	2(0.01)	0(0.00)	2(0.01)	1.602	0.206

## Discussion

In this study, we calculated the monthly positivity rate of respiratory pathogens among hospitalized children and employed Joinpoint regression analysis to explore the temporal trends in pathogen positivity rates.Monthly positivity rates of respiratory pathogens showed dynamic changes. These patterns reflect complex interactions among multiple factors influencing pathogen transmission From January to April 2020, the pathogen positivity rate significantly declined (monthly percentage change, MPC = -41.57), a phenomenon likely closely associated with the stringent COVID-19 prevention and control measures implemented at the time, including lockdown policies, travel restrictions, and enhanced hygiene practices. These non-pharmaceutical interventions (NPIs) not only effectively suppressed the transmission of SARS-CoV-2 but also significantly reduced the spread of other respiratory pathogens [23]. However, from then until September 2020, the pathogen positivity rate exhibited a rapid upward trend (MPC = 37.62), attributable to the gradual relaxation of control measures and the seasonal relapse of respiratory viruses. From September 2020 to January 2023, the pathogen positivity rate showed a gradual downward trend (monthly percentage change, MPC = -2.31), likely influenced by a combination of factors, including the sustained implementation of public health measures, seasonal fluctuations in pathogen activity, and partial development of herd immunity. By September 2023, the pathogen positivity rate rebounded again (MPC = 10.99) and subsequently continued to exhibit a slow downward trend until December 2024 (MPC = 1.67), indicating persistent fluctuations in the transmission of respiratory pathogens. The research findings from Huili Hospital on Prunus salicina also revealed three significant turning points in pathogen activity [24]. The four significant turning points in pathogen activity identified in this study not only highlight the dynamic characteristics of respiratory pathogen epidemiology but also further corroborate the dual influence of external intervention measures and natural disease transmission patterns.

Our study revealed a persistent phenomenon: the positivity rate of respiratory pathogens exhibited a significant male predominance, a trend that remained unchanged both during the pandemic and in the post-pandemic period. This aligned with global reports indicating increased infection risks among male children [6–9]. This phenomenon might have stemmed from the regulatory role of X chromosome-linked immune genes and delayed mucosal immune maturation [25], combined with greater outdoor exposure among boys. Notably, HRV and HRSV infections also demonstrated consistent male predominance, potentially associated with higher expression levels of viral entry receptors (e.g., ICAM-1 for HRV) in male airway epithelial cells [26].

Pathogen positivity rates increased across all age groups in the post-pandemic period. Concurrently, the distribution of pathogens shifted significantly among age groups. Concurrently, significant alterations in the distribution of pathogens among age groups indicated that the stringent non-contact preventive measures enforced during the pandemic had enduring effects on the population’s immune exposure patterns. HRV detection rates remained consistently elevated across all age groups, suggesting HRV’s diminished susceptibility to non-pharmaceutical interventions (NPIs)—a conclusion that aligns with a New Zealand study [27]. This may be attributed to HRV’s non-enveloped viral structure, which confers strong environmental stability and transmission capabilities, rendering it resistant to disinfectants such as alcohol and allowing for prolonged survival on surfaces [27–28]. HRSV positivity rates were notably higher in infants and young children compared to other age groups, likely due to their immature mucosal immunity and lack of prior exposure, which increases their susceptibility [18, 23–24]. Preschool-aged children exhibited distinct changes: elevated Mp and HAdV positivity rates coincided with decreased HRSV and HRV positivity, indicating potential pathogen competition or age-related immune maturation [24, 29]. School-aged children displayed the highest Mp positivity rate, consistent with previous studies [18, 24]. Chi-square trend analysis further confirmed that Mp positivity rates increased significantly with age, while HRSV exhibited a pronounced decline, corroborating earlier research [29–30], and highlighting significant age-related differences in susceptibility to various pathogens. Infants and young children were predominantly infected by viruses, and as age progresses, the proportion of atypical pathogens, such as Mp infections, gradually increases.

From 2020 to 2022, the transmission of influenza viruses was nearly absent, with only brief and mild transmission peaks observed. This trend aligns with findings from China’s Shenzhen [7] and Mexico [31]. However, during the autumn and winter of 2021, the FluB resurged, experiencing surges from November 2023 to April 2024. The influenza A virus exhibited minor fluctuations between May and September 2022, with surges occurring from March to May 2023 and from October 2023 to May 2024. This typical bimodal pattern of high prevalence in spring and winter is also observed in northern China [32]. The aforementioned observations suggest that the pandemic and NPIs have disrupted the traditional seasonal patterns of influenza transmission. Enhancing epidemiological surveillance of influenza can aid in mitigating potential public health crises.

The overall trend of HAdV remained relatively stable from 2020 to 2023; however, a significant surge began in May 2024. The peak occurred between May and July, with the positive rate reaching 26.80% in June. This explosive growth was also observed in Guangdong Province [16]. Additionally, HAdV-related respiratory infections emerged in Vietnam [33] and the United States [34] during the later stages of the pandemic. Analysis suggests that the COVID-19 pandemic and NPIs may have prevented the widespread transmission of HAdV, while also reducing children’s immunity to adenoviruses. This heightened susceptibility, combined with the absence of herd immunity, led to a significant rise in the HAdV positivity rate in 2024.The transmission of HRV and HBoV showed no significant changes and remained detectable. Notably, HRV maintained a consistently high positive rate without distinct seasonal patterns.

Previous studies indicate that Mp outbreaks occur every 3–7 years, lasting 1–2 years [35–36]. A Beijing study revealed that increased Mp detection began in the summer of 2019, following a sharp decline in Mp positivity rates after the COVID-19 pandemic emerged in late 2019 [36]. Post-pandemic, global Mp incidence dropped from 8.61% to 1.69% in 2020 [37]. During our study period (2020–2022), Mp remained at low incidence. However, since April 2023, MP infection rates have surged, with positivity rates exceeding 50% by November. Although 2024 saw a slight decline, rates still remained higher than those during 2020–2022. A Spanish study showed that 2023 positivity rates were significantly higher compared to 2020–2022. In addition, similar changes were observed in China’s northeast and Inner Mongolia [38], as well as in Shanghai [39], with an Mp epidemic peak occurring in 2023. The decline in infection rates during the pandemic period may be related to NPI, while the resurgence after 2023 aligned with its epidemiological patterns. It is important to note a significant limitation regarding Mp detection. In this study, Mp was detected using multiplex RT-PCR from throat swab samples. This molecular method detects Mp DNA but cannot distinguish between asymptomatic colonization and active infection. Mp is known to colonize the respiratory tract of children without causing disease, and the prevalence of asymptomatic carriage varies by age and geographic region. Therefore, the observed increase in Mp detection after 2023 should be interpreted as an increase in Mp positivity rates or enhanced detection rather than definitive evidence of Mp disease outbreaks or epidemics. The clinical significance of these findings would require correlation with clinical symptoms, radiological findings, and serological evidence of acute infection, which were not available in this retrospective laboratory-based study. RSV showed atypical seasonal peaks in May to September 2023 and February to April 2024, which deviated from previous winter patterns. Studies in Australia also indicated atypical seasonal peaks during the summer of 2023 [40]. Additionally, HMPV, HPIV, and HCoV exhibited atypical seasonal fluctuations.

Compared to the pandemic period, the co-infection rate in the post-pandemic period increased from 5.28% to 12.27%. The predominant combinations of co-infections shifted from HRV with HRSV, HPIV, and HADV to HRV + Mp, HRV + HADV, and HADV + Mp, though HRV remained the most prevalent pathogen in co-infections. A study by Shenzhen Children’s Hospital revealed that the co-infection rate of pediatric respiratory pathogens rose from 6.3% in 2020–2022 to 15.1% in 2023–2024 [7]. These findings indicate a significant increase in co-infection rates following the full relaxation of non-pharmaceutical interventions. While some studies suggest pathogen interference, the underlying mechanisms remain unclear [41–42]. Previous studies have reported that co-infections may be associated with more severe clinical outcomes and increased medical burden [43–44]. However, our retrospective laboratory-based study did not collect clinical data such as disease severity, ICU admission, or length of stay. Therefore, we cannot draw conclusions about the clinical impact of co-infections in our study population. Future studies incorporating detailed clinical correlates are needed to determine whether the observed increase in co-infection rates translates into greater disease severity or healthcare utilization.

This study has several limitations. First, its single-center design and exclusive focus on hospitalized pediatric patients may limit the generalizability of the findings to the general pediatric population. The pathogen positivity rates reported in this study reflect laboratory detection among hospitalized pediatric patients with acute respiratory infections, rather than true community incidence or prevalence. During the COVID-19 pandemic, hospitalization thresholds and healthcare-seeking behaviors underwent substantial changes due to various factors, including more stringent hospitalization criteria implemented by healthcare facilities to reduce nosocomial transmission risks, parental fear of contracting SARS-CoV-2 in hospital settings, and restricted access to medical services during lockdown periods. These factors likely resulted in more severe cases being hospitalized during the pandemic period, potentially affecting the observed pathogen detection rates. Conversely, in the post-pandemic period, relaxed restrictions and increased public awareness may have led to earlier hospitalization for less severe cases. These dynamic changes in healthcare-seeking behavior and hospitalization criteria should be considered when interpreting the observed differences in pathogen positivity rates between the two periods.Second, the analysis was restricted to 11 pathogens and lacked integration with detailed clinical data, including disease severity scores, intensive care unit admission, length of hospital stay, and mortality. This confines the epidemiological scope. Third, the study period, while covering key pandemic and post-pandemic phases, is relatively limited. Future multi-center studies incorporating a broader range of pathogens, clinical correlates, and extended surveillance are needed to continuously monitor epidemiological trends and better inform public health strategies.

## Conclusions

Among 59,526 hospitalized children with ARIs who met inclusion criteria and underwent complete pathogen testing, the overall pathogen positivity rate was 60.2%, with HRV, Mp, and HRSV being predominant. Pathogen distribution varied significantly by gender, age, and season. Marked epidemiological shifts occurred in the post-pandemic period, characterized by increased overall positivity and mixed infection rates. Successive increases of FluA, FluB, Mp, and HAdV were observed post-pandemic. Continuous surveillance and in-depth understanding of these patterns are essential for effective respiratory infection control. However, future studies incorporating clinical outcomes and a broader patient spectrum are needed to fully characterize the post-pandemic epidemiological landscape.

## Data Availability

The datasets used in this study are available upon request from the corresponding author.

## Abbreviations

COVID-19: Coronavirus Disease 2019
Mp: Mycoplasma pneumoniae
FluA: Influenza A
FluB: Influenza B
HAdV: Human adenovirus
HBoV: Human bocavirus
HPIV: Human parainfluenza virus
Ch: Chlamydia
HMPV: Human metapneumovirus
HCoV: Human coronavirus
HRV: Human rhinovirus
HRSV: Human respiratory syncytial virus
ARI: Acute respiratory infections
NPI: Non-pharmaceutical interventions
